# Marine Organisms for the Sustainable Management of Plant Parasitic Nematodes

**DOI:** 10.3390/plants10020369

**Published:** 2021-02-14

**Authors:** Pasqua Veronico, Maria Teresa Melillo

**Affiliations:** Institute for Sustainable Plant Protection, CNR, Via G. Amendola 122/D, 70126 Bari, Italy; mariateresa.melillo@ipsp.cnr.it

**Keywords:** plant-parasitic nematodes, seaweeds, marine cyanobacteria, sponges, sustainable strategies

## Abstract

Plant parasitic nematodes are annually responsible for the loss of 10%–25% of worldwide crop production, most of which is attributable to root-knot nematodes (RKNs) that infest a wide range of agricultural crops throughout the world. Current nematode control tools are not enough to ensure the effective management of these parasites, mainly due to the severe restrictions imposed on the use of chemical pesticides. Therefore, it is important to discover new potential nematicidal sources that are suitable for the development of additional safe and effective control strategies. In the last few decades, there has been an explosion of information about the use of seaweeds as plant growth stimulants and potential nematicides. Novel bioactive compounds have been isolated from marine cyanobacteria and sponges in an effort to find their application outside marine ecosystems and in the discovery of new drugs. Their potential as antihelmintics could also be exploited to find applicability against plant parasitic nematodes. The present review focuses on the activity of marine organisms on RKNs and their potential application as safe nematicidal agents.

## 1. Introduction

Plant-parasitic nematodes (PPNs) are one of the most important agricultural pests. Thousands of crops and trees are susceptible to nematode infections, which result in 10%–25% global yield losses [1,2]. Most economic losses are caused by sedentary PPN genera, principally the root-knot (*Meloidogyne* spp.) and the cyst (*Heterodera* spp. and *Globodera* spp.) nematodes [3]. Root-knot nematodes (RKNs) constitute a major group of PPNs that are distributed worldwide. They are obligate root parasites of thousands of plant species and damage both horticultural and field crops [4]. Infection causes root galls and hinders the normal uptake of water and nutrients, resulting in poor growth, loss of quality and yield and reduced resistance to other biotic and abiotic stresses [5]. The economic impact of these parasites is expected to further increase due to global climate change and the resulting occurrence of new invasive nematode species and the emergence of virulent populations able to overcome plant resistance genes [6,7]. Control of RKNs has been achieved mainly through soil treatment with chemical nematicides, whose use has been severely restricted by recent European legislation and subsequent additions (Reg. EC 1107/2009; 459/2010 and 293/2013), which focus on animal and human health as well as the environment. The total ban or restricted use of most nematicides has raised an urgent need for safe and effective control options [8], thus increasing research on sustainable alternatives such as biocontrol agents, green manures and organic amendments [9,10,11,12]. Compounds deriving from plants, microorganisms and marine organisms could ensure a virtuous combination of nematicidal efficacy and environmental safety, as their natural origin ensures their low persistence both in soil and crops and, therefore, a low impact on the environment and human and animal health [13,14]. Due to their distribution in different ecosystems and their accessibility, plants constitute a major source of specialized metabolites suitable for crop parasite and pathogen control. Much effort has been put into developing new bioactive agents from plants [15,16,17]. However, with oceans covering more than 70% of the Earth’s surface, marine biodiversity may offer an alternative source of useful specialized metabolites for the control of plant, animal and human parasites and pathogens. Marine organisms have already yielded thousands of chemicals, with hundreds of new compounds still being discovered every year. These have yielded several bioactive metabolites that have been successfully developed by the pharmaceutical industry—mainly for human diseases [18,19,20]. However, several marine natural products abundantly present in microorganisms, algae and invertebrates can also represent a source of potentially exploitable nematicidal compounds for the formulation of new, safe nematicides. This review will focus on advances in the roles played by natural compounds from marine environments in the control of plant-parasitic nematode infestation.

## 2. Seaweeds

Algae were primarily known as growth stimulants due to their positive effects on plant photosynthetic pigments and on the content of total carbohydrates, starch, amino acids and proteins [21]. Moreover, the application of seaweed extracts can stimulate nitrogen uptake and metabolism in the treated plants [22]. The stimulatory effect of marine bioactive substances has been ascribed to the presence of phytohormones such as auxins, gibberellins and the precursor of ethylene and betaine, together with many other organic and inorganic molecules [23]. Algae synthesize unusual and complex polysaccharides such as agars, alginates, carrageenans, fucans and phlorotannins, which show a variety of biological activities and are involved in host defense mechanisms [23,24].

Interest in the application of seaweed extracts in the management of PPNs, especially of RKNs, has increased in the last decade due to the combination of their beneficial effects on plants and their activity towards several plant pathogens. Seaweed extract applications on plants result in improved crop performance and yield, early seed germination and elevated resistance to biotic and abiotic stresses [24,25]. Improvement in diving and cultivation methods, as well as in the recycling of seaweed wastes, may help to disclose the huge potential of these marine organisms for sub-tropical and tropical root-knot nematode management. Table 1 summarizes the seaweed that was exploited during in vitro and in vivo studies, as well as the details of the trial, material, dose and type of inoculum that was used.

### 2.1. Seaweed’s Effect on Juvenile Viability and Egg Hatching

Egg hatching, motility and survival are critical behaviors in the RKN life cycle. Interference in any of these steps could interrupt the nematode’s intact life cycle and effectively control its reproduction and ability to damage the host plant [26].

Several studies reported nematostatic and nematicidal activity of seaweed extracts, which causes inhibition of egg hatching and/or induction of second-stage juvenile (J2) mortality (Table 1). However, studies on seaweeds from phyla Phaeophyta (brown algae), Rhodophyta (red algae) and Chlorophyta (green algae) have also indicated that not all marine algae have a nematicidal activity.

The highest nematicidal potential has generally been demonstrated by species of marine brown seaweeds. In a screening of eighteen species of marine algae, Paracer et al. [27] reported that only the aqueous extract of the brown alga *Spatoglossum schroederi* killed J2 of *Meloidogyne incognita*, *Meloidogyne javanica* and *Meloidogyne arenaria* after a 24 h exposure to concentrations of 1.0, 0.75, and 0.50% (dry weight in aqueous solutions). They also related this nematicidal efficacy to extract acidity as larvae appeared vacuolated within 24 h. A strong nematicidal activity against *M. javanica* J2 was also displayed by aqueous, ethanol and methanol extracts of the brown alga *Stoechospermum polypodioides* [28,29,30,31] and by the extracts of the brown seaweeds *Sargassum tenerrimum*, *Sargassum swartzii* and *Sargassum wigbtii* [32]. In a further in vitro study on the effect of 32 seaweeds on *M. javanica* J2 mortality and egg hatching, a 72 h exposure of J2 and eggs to water and methanol extracts from Phaeophyta species *S. tenerrimum*, *Padina tetrastromatica* and *Melanothamnus afaqhusainii* caused an almost complete J2 mortality and egg hatching suppression [33].

In general, marine brown algae always exhibit a stronger nematicidal activity against juveniles when compared with green and red seaweeds. In a comparative assay on *M. javanica* J2 viability and egg hatchability, extracts from brown seaweeds (*S. tenerrimum*, *Cystoseira indica*, *Jolyna laminarioides*, *P. tetrastromatica*, and *Lyengaria stellata*) were found to be highly active on nematode J2 as they caused complete mortality after a 72 h exposure and strongly reduced egg hatching (4% vs. 68% of the nontreated control) [34]. Conversely, the green algae *Ulva rigida* and *Codium iyengarii* were less effective as they caused only 30 and 35% J2 mortality and 30 and 26% egg hatching, respectively [34]. Similarly, 90%–97% mortality of *M. incognita* J2 occurred after a 72 h exposure to 2.5, 5 and 10% extracts from the brown algae *Cystoseria myrica*, *Cystoseria trinode* and *Padina pavonia*, whereas a lower activity (73% mortality) was recorded for a similar treatment with the extract from the red alga *Digenea simplex* [35].

By contrast, ethanol extracts of the brown alga *Stokeyia indica*, green alga *C. iyengarii* and red seaweeds *Jania capillacea* and *Solieria robusta* showed similar activities on *M. javanica* J2s and caused more than 50% mortality rates after a 24 h exposure to a 10 mg mL^−1^ concentration [36]. A comparison of the effects on *M. incognita* J2 and eggs of aqueous extracts from the green algae *Enteromorpha flexuosa*, *Ulva lactuca* and *Codium fragile*, the brown algae *C. myrica* and *Sargassum muticum* and the red algae *Dilsea carnosa* and *Laurencia nidifica* showed the highest J2 mortality (86.7%) for the extracts of *C. fragile*, *D. carnosa* (82.9%) and *C. myrica* (73.3%), whereas the lowest egg hatching percentages were recorded for egg treatment with extracts of *E. flexuosa*, *D. carnosa* and *C. fragile*—15.9, 20.6 and 22.7%, respectively [37]. Finally, a comparative in vitro experiment with ethanol extracts of the red algae *Corallina mediterranea* and *Corallina officinalis* and the green alga *Ulva fasciata* indicated a higher effect of *U. fasciata* extract on *M. incognita* egg hatching (an 87% egg hatching decrease after a 3-day exposure), whereas all extracts were similarly active on J2 and caused 80%–85% mortality rates after a 12 h exposure to 0.5 or 1 mg mL^−1^ concentrations [38].

In spite of the large number of studies on seaweed raw extracts, there are very few commercial formulations of these products and their effectiveness on root-knot nematodes is poorly documented. In a study on the effects of two commercial formulations of the brown algae *Ascophyllum nodosum* and *Ecklonia maxima* on the root-knot species *Meloidogyne chitwoodi* and *Meloidogyne hapla*, Ngala et al. [39] reported a significant effect on *M. chitwoodi* egg hatching and J2 infectivity for only the *A. nodosum* extract. It has been assumed that compounds of *A. nodosum* extract can interact with enzymes, such as lipase, proteinase and chitinase, involved in the hatching process [40].

### 2.2. Seaweed Effects on Nematode Reproduction and Multiplication and Plant Growth

Several reports have indicated that the application of seaweed extracts or biomasses to soil can result in decreased levels of nematode attack and improved plant growth (Table 1).

The first investigations of seaweed nematicidal effects in soil date back to last century and are focused mainly on the brown alga *A. nodosum*. Tarjan [41] found that the application of *A. nodosum* extracts to citrus seedlings infested by *Radopholus similis* caused a significant reduction in nematode density and increased plant weight when compared with non-treated plants. Likewise, commercial extracts of *A. nodosum* (Maxicrop and Sea-Born), when applied to the soil, controlled *Belonolaimus longicaudatus* on centipede grass [42]. Following these first reports, a number of studies demonstrated a significant suppressive effect of *A. nodosum *extracts on root-knot nematodes—mainly *M. incognita* and *M. javanica* on tomatoes [43,44,45,46]. These studies unanimously reported a reduced root-knot nematode infestation and number of eggs, as well as improved plant growth, following soil treatments with *A.* *nodosum* extracts. Nour El-Deen et al. [47] documented that the treatment of basil plants with the combined application of *A. nodosum* extract, cabbage (*Brassica oleracea*) leaves and arabic gum, resulted in the highest reduction of *M. incognita* galls and egg masses and in an increased plant fresh weight. Wu et al. [44] suggested that effects of *A. nodosum* extract on nematodes are at least partially due to the betaine content of the extract. In this respect, further studies by the same authors [45] indicated that betaines were absorbed by plants and stimulated an increased resistance to nematode invasion. In the same study, *Arabidopsis thaliana* plants grown under monoxenic conditions in the presence of an alkaline extract of *A. nodosum* or a mixture of betaines (γ-aminobutyric acid betaine; δ-aminovaleric acid betaine; glycinebetaine) exhibited a significant decrease in *M. javanica* females in plant roots and in the number of eggs recovered 45 days after inoculation. Finally, soil treatments with *A. nodosum* were also found to be effective in controlling *M. chitwoodi* infestation on tomato plants and in improving the growth of nematode-infested plants [39].

Featonby-Smith and van Staden [48] tested the effects of another brown alga, *E. maxima*, and reported that treatment with seaweed concentrate (Kelpak 66, Kelp Products (Pry.) Ltd., Cape Town, South Africa) reduced *M. incognita* galling and induced the growth of tomato roots. Although the number of nematodes increased in the soil after the application of seaweed concentrate, the numbers that had established themselves in the roots were reduced when compared with the control. Presence of cytokinins in marine algae extracts may be responsible for nematicidal activity as it has been stated that high concentrations of kinetin inhibited both larval penetration and development in the roots of tomato plants [49]. Modes of application of *E. maxima* seaweed concentrate (SWC) resulted in the opposite effects [50]. Foliar-applied SWC did not enhance plant growth and encouraged nematode galling, whereas a significant reduction in nematode infestation was noted when SWC was applied as a soil drench. The effectiveness of the seaweed products from *E. maxima* was also demonstrated by the control of *M. chitwoodi* and *M. hapla* infections on tomato plants [39].

As in the in vitro studies described above, brown algae showed a higher nematicidal effectiveness when compared with the red and green algae in in vivo experiments. Soil treatments with the aqueous extracts of the brown algae *C. myrica*, *C. trinode* and *P. pavonia* were found to be more effective in reducing the *M. incognita* infestation on eggplants than the extracts from the red alga *D. simplex*, the green alga *C. serrulata* and the seagrass *Thalassodendron ciliatum*, though the beneficial effects of all extracts were improved by their combination with chemical nematicides [35].

Soil drench treatments (1 mg/mL) with ethanolic extracts of the green alga *U. fasciata* Delile and the red algae *C. officinalis* and *C. mediterranea* significantly reduced *M. incognita* infestation on tomato plants, though the extract of *U. fasciata* was much more effective (a 77.5% reduction in gall number/root) than the *C. officinalis* and *C. mediterranea* extracts, with a 50.3 and 34.7% reduction, respectively [38,51].

As indicated by another large group of studies, an alternative option for the exploitation of seaweeds in nematode control is represented by soil amendments with raw or composted seaweed biomasses. Soil treatments with dry powder of the brown alga *J. laminarioides* and the red algae *Cystoclonium purpereum* and *Hypnea valentiae* resulted in a strong reduction of root-gall formation on okra and tomato roots infested by *M. javanica* and in improved plant growth [34]. In a study of El-Ansary and Hamouda [52], the soil application of powdered dry biomass from *U. lactuca* (green alga), *Jania rubens* and *Laurencia obtuse* (red seaweeds) and the brown alga *Sargassum vulgare* significantly decreased galls and root-knot nematode egg masses on banana (*Musa* spp.) roots and the nematode population in soil. The efficacy of *U. lactuca* can be explained by the high content of nematotoxic phenolics. Analogously, soil amendments with dry powder of *S. tenerrimum* significantly reduced root-knot nematode infestation on okra roots [32,34]. The combination of the brown seaweed *Stoechospermum marginatum* and biocontrol agents resulted in the effective control of *M. javanica* on okra [53]. In addition, soil amendments with the dry powder of the red alga *M. afaqhusainii* significantly reduced the *M. javanica* penetration in sunflower roots [54] and controlled the *M. javanica* infestation in okra plants [55]. Finally, soil amendments with dry powdered biomasses from *S. swartzii*, *Spatoglossum asperum*, *Spatoglossum variabile* and *S. schroederi* were repeatedly documented as being effective at controlling various root-knot nematodes on tomatoes. They also enhanced plant growth and yield [27,54,56]. A key issue for the commercial exploitation of natural nematicidal compounds is represented by the definition of technical formulations as being able to prolong their shelf life and ensure a feasible field application. In this regard, vehiculation by alginates and green silver nanoparticles has been the technical approach most frequently investigated for algal extracts.

Alginate is a polysaccharide extracted from marine brown algae that is generally used as a gelling agent in food and in the pharmaceutical industry, as well as for the formulation of biocontrol agents [57,58,59]. The main advantages of alginate preparations are the absence of any toxicity and quick degradability in soil. A recent greenhouse study of El Ansary et al. [60] documented a significant reduction of the *M. javanica* infestation in eggplants following soil treatments with algal alginates from *Colpomenia sinuosa*, *Turbinaria turbinate* and *C. myrica*. This peaked at an almost 80% reduction in egg formation on eggplant roots. It has been suggested that the ability of alginates to control nematode infection relies on triggering plant defense responses, probably through the salicylic acid signaling pathway [61].

In recent years, nanoparticles such as nanosilvers have been proposed as a new tool for the control of plant pathogens and parasites—including nematodes [62,63,64]. Under greenhouse conditions, soil treatments with green silver nanoparticles synthesized from the extracts of *U. lactuca* and *T. turbinate* resulted in a significant reduction in galls, *M. javanica* females, egg-masses on the eggplant root systems and juveniles as well as in a remarkable increase in plant growth [65].

## 3. Cyanobacteria

Cyanobacteria are prokaryotic oxygenic phototrophs that are found in almost every habitat on earth. Among the five divisions of Cyanobacteria, Cyanophyta (blue-green algae) and Pyrrophyta (dinoflagellates) are rich sources of novel compounds and have been extensively investigated [19,66].

Several studies have unraveled the nematicidal potential of blue-green algae from fresh water and soil species [67]. Pioneer investigations documented an increased plant-parasitic nematode mortality and/or hatching inhibition following the inoculation of soil with endospores [68,69] or extracts and exudes [70,71] from cyanobacteria. Moreover, root-knot nematode egg masses and galls were also reduced by the incorporation into soil of blue-green algae [72,73,74,75].

Fresh water blue-green algae species exhibit an easier cultivability in comparison with marine organisms. However, the advancement in cultivation technologies and in molecular biology techniques has raised a renewed interest in exploring the marine habitat for new drugs. Marine forms of cyanobacteria are rich sources of metabolites and toxins, which are potentially novel bioactive compounds with wide pharmaceutical applications, especially towards the treatment of many human diseases. The marine cyanobacteria *Lyngbya* spp. are, to date the most productive source of bioactive cyanobacterial compounds [76] and show potent anti-oxidant, anti-inflammatory, anti-diabetic and anti-cancer activity [19,77]. In particular, lipopeptides isolated from the cyanobacterium *Lyngbia majuscula* have shown a spectrum of biological activities consistent with their functioning as defense metabolites, including sodium channel blocking activity and arthropod and fish toxicity [78,79]. The nematicidal potential of these compounds is almost completely uninvestigated as, to the best of our knowledge, only one assay of the activity of a *Microcoleus lyngbyaceus* extract against *M. incognita* has been performed; however, it resulted ineffective at the applied concentrations [27].

## 4. Sponges

Sponges are an important source of biologically active natural products [80]. However, sponges are often associated with symbiotic microbial populations such as: archaea, bacteria, actinomycetes, fungi, cyanobacteria and microalgae, which represent the probable source of bioactive metabolites [81]. The strain BCPBMS-1 of *Pseudomonas fluorescens*, isolated from the marine sponge *Callyspongia diffusa*, showed nematicidal activity against *M. javanica* both in vitro and in pot experiments [82].

To the best of our knowledge, no other studies have been carried out to unravel the biocontrol potential of sponges or their associated microorganisms against PPN, though several products isolated from marine sponges [80] demonstrated an anthelmintic activity against *Haemonchus contortus*, a socioeconomically important parasitic nematode of livestock animals (Table 2). Amphilactams A–D, isolated from an *Amphimedon* sp. sponge, inhibited *H. contortus* larval development but had poor or nil activity against nematode eggs [83]. A strong inhibition of larval development of the same nematode was also reported for: polyketides geodin A, isolated from the sponge *Geodia* sp. [84], onnamide F (2), isolated from the marine sponge *Trachycladus laevispirulifer* [85] and the 6-N-acyladenine phorioadenine A, which was detected in the crude extract from the *Phoriospongia* sp. (CMB-03107) [86]. Finally, a moderate to limited inhibition of *H. contortus* motility was recently documented for fromiamycalin, a pentacyclic guanidine alkaloid, and halaminol A, an acetamide that were isolated from the extracts of the demosponges *Monanchora unguilata* and *Haliclona* sp., respectively [87].

## 5. Conclusions

Literature reports indicate that marine organisms such as seaweeds, cyanobacteria and sponges can be a huge source of new nematicidal products. Seaweeds seem to offer the highest potential of exploitation. They have largely proved to have a high effectiveness for relevant phytonematode species, as well as for plant bio-stimulating effects. Information about the mechanisms of nematicidal activity and the plant growth-promoting effects of seaweeds is almost nil. This shows that more in-depth studies are required to elucidate their role in direct nematode control and plant resistance to nematode attack. Phenotyping of various seaweed extracts could be a useful tool to assess their bioactive components, application rates, timing and genetic variation in plant responses. Cyanobacteria and sponges also represent promising raw materials for new nematicides, though further study is required. The keystone for the industrialization of these products and of most natural nematicides, would be the development of innovative formulations such as micro or nanoencapsulation in order to preserve product bioactivity and ensure technical feasibility.

As plant-derived compounds and biocontrol agents, marine-product-derived nematicides represent a helpful tool for nematode management in organic systems. However, use on conventional crops should be limited to low nematode infestations or in combination with synthetic nematicides.

## Figures and Tables

**Table 1:** Seaweeds tested for the control of plant-parasitic nematodes.

Organism	Nematode	Type of Study	Material	Dose	Inoculum	Host Plant	Reference
**Phaeophyta**							
*Ascophyllum nodosum*	*M. javanica*	In vitro, in vivo (P)	Maxicrop	3.6%	J2	Tomato	[43]
	*M. javanica*	In vivo (P)	Maxicrop	3.6%	J2	Tomato	[44]
	*M. javanica*	In vivo (A)	Maxicrop	0.075%	J2	Arabidopsis	[45]
	*M. incognita*	In vivo (P)	Algaefol	10–25 mL/Kg soil	eggs	Tomato	[46]
	*M. javanica*	In vivo (P)	extract	1%	eggs	Basil	[47]
	*M. chitwoodi*, *M. hapla*	In vitro, in vivo (P)	OSMO	5 mL/L	J2	Tomato	[39]
	*Radopholus similis*	In vivo (P)	pwd extract	2.24 Kg/ha	NI	Citrus	[41]
	*Belonolaimus longicaudatus*	In vivo (F)	Maxicrop, Seaborn	2.25–4.5–6.75 Kg/ha 2.47–4.94–7.42 L/ha	NI	Centipede grass	[42]
*Colpomenia sinuosa*	*M. javanica*	In vivo (P)	dry alginate	2.5 g/100 mL	J2	Eggplant	[60]
*Cystoseria indica*	*M. javanica*	In vitro					[34]
*Cystoseria myrica*	*M. incognita*	In vitro					[35,37]
	*M. incognita*	In vivo (P)	dry powder	20 g/plant	J2	Eggplant	[35]
	*M. javanica*	In vivo (P)	dry alginate	2.5 g/100 mL	J2	Eggplant	[60]
*Cystoseria trinode*	*M. incognita*	In vitro, in vivo (P)	dry powder	20 g/plant	J2	Eggplant	[35]
*Ecklonia maxima*	*M. incognita*	In vivo (P)	Kelpak 66	1:500 *v*/*v*	J2	Tomato	[48]
	*M. incognita*	In vitro, in vivo (P)	Kelpak	0.2–0.4–1%	NI	Tomato	[50]
	*M. chitwoodi*, *M. hapla*	In vitro, in vivo (P)	Kelpak	10 mL/L	J2	Tomato	[39]
*Lyengaria stellata*,	*M. javanica*,	In vitro					[34]
*Jolyna laminarioides*,	*M. javanica*,	In vitro, in vivo (P)	dry powder	1% *w*/*w*	eggs	Okra, Tomato	[34]
*Padina pavonia*	*M. incognita*	In vitro, in vivo (P)	dry powder	20 g/plant	J2	Eggplant	[35]
*Padina tetrastrommatica*	*M. javanica*	In vitro					[33,34]
*Sargassum muticum*	*M. incognita*	In vitro					[37]
*Sargassum tenerrimum*	*M. javanica*	In vitro					[32,33,34]
	*M. javanica*	In vivo (P)	dry powder	0.5–1% *w*/*w*	eggs	Okra	[32,34]
*Sargassum swartzii*	*M. javanica*	In vitro					[32]
	*M. javanica*	In vivo (P)	dry powder	1% *w*/*w*	Eggs	Okra	[32,34]
	*M. javanica*	In vivo (P)	dry powder	0.5–1% *w*/*w*	Eggs/J2	Tomato	[54,56]
*Sargassum vulgare*	*Meloidogyne* spp.	In vivo (P)	dry powder	5 g/kg soil	J2	Banana	[52]
*Sargassum wigbtii*	*M. javanica*	In vitro					[32]
	*M. javanica*	In vivo (P)	dry powder	1% *w*/*w*	eggs	Okra	[32,34]
*Spatoglossum asperum*	*M. javanica*	In vivo (P)	dry powder	0.5–1% *w*/*w*	J2	Tomato	[56]
*Spatoglossum variabile*	*M. javanica*	In vivo (P, F)	dry powder	1% *w*/*w*; 70 g/2 m row	eggs/J2	Sunflower, Tomato	[54]
*Spatoglossum schroederi*	*M. incognita*, *M. javanica*, *M. arenaria*	In vitro, in vivo (P)	dry powder	1% *w*/*w*	eggs	Tomato	[27]
*Stoechospermum marginatum*	*M. javanica*	In vivo (P)	dry powder	n.a.	eggs	Okra	[53]
*Stoechospermum polypodioides*	*M. javanica*	In vitro					[28,29,30,31]
* Stokeyia indica *	*M. javanica*	In vitro					[36]
*Turbinaria turbinate*	*M. javanica*	In vivo (P)	dry alginate nanoparticles	2.5 g/100 mL 4.25–8.5–12.75–17 mg/100 mL	J2 J2	Eggplant	[60,65]
**Chlorophyta**							
*Caulerpa serrulata*	*M. incognita*	In vitro, in vivo (P)	dry powder	20 g/plant	J2	Eggplant	[35]
*Codium fragile*	*M. incognita*	In vitro					[37]
*Codium iyengarii*	*M. javanica*	In vitro					[34,36]
*Enteromorpha flexuosa*	*M. incognita*	In vitro					[37]
*Halimeda tuna*	*M. javanica*	In vivo (P, F)	dry powder	1% *w*/*w*; 70 g/2 m row	eggs/J2	Sunflower, Tomato	[54]
*Ulva fasciata*	*M. incognita*	In vitro, in vivo (P)	extract	1 mg/mL	Eggs	Tomato	[51]
	*M. incognita*	In vitro, in vivo (P)	extract	1 mg/mL	J2	Tomato	[38]
*Ulva lactuca*	*M. incognita*	In vitro					[37]
	*Meloidogyne* spp.	In vivo (P)	dry powder	5 g/kg soil	J2	Banana	[52]
	*M. javanica*	In vivo (P)	dry alginate	2.5 g/100 mL	J2	Eggplant	[65]
*Ulva rigida*	*M. javanica*	In vitro					[34]
**Rhodophyta**							
*Corallina mediterranea*	*M. incognita*	In vitro, in vivo (P)	extract	1 mg/mL	Eggs	Tomato	[51]
	*M. incognita*	In vitro, in vivo (P)	extract	1 mg/mL	J2	Tomato	[38]
*Corallina officinalis*	*M. incognita*	In vitro, in vivo (P)	extract	1 mg/mL	Eggs	Tomato	[51]
	*M. incognita*	In vitro, in vivo (P)	extract	1 mg/mL	J2	Tomato	[38]
*Cystoclonium purpereum*	*M. javanica*	In vitro, in vivo (P)	dry powder	1% *w*/*w*	eggs	Okra, Tomato	[34]
*Digenea simplex*	*M. incognita*	In vitro, in vivo (P)	dry powder	20 g/plant	J2	Eggplant	[35]
*Dilsea carnosa*	*M. incognita*	In vitro					[37]
*Hypnea valentiae*	*M. javanica*	In vitro, in vivo (P)	dry powder	1% *w*/*w*	eggs	Okra, Tomato	[34]
* Jania capillacea * ,	*M. javanica*	In vitro					[36]
* Jania rubens *	*Meloidogyne* spp.	In vivo (P)	dry powder	5 g/Kg soil	J2	Banana	[52]
* Laurencia nidifica *	*M. incognita*	In vitro					[37]
* Laurencia obtuse *	*Meloidogyne* spp.	In vivo (P)	dry powder	5 g/Kg soil	J2	Banana	[52]
*Melanothamnus afaqhusainii*	*M. javanica*	In vitro					[33,34]
	*M. javanica*	In vivo (P, F)	dry powder	1% *w*/*w*; 70 g/2 m row	eggs/J2	Sunflower, Tomato	[54]
	*M. javanica*	In vivo (P)	dry powder	0.25–0.5–1% *w*/*w*	eggs	Okra	[55]
* Solieria robusta *	*M. javanica*	In vitro					[36]

P = pots; F = field; A = agar plates; NI = natural infested soil; pwd extract = powdered extract.

**Table 2:** Anthelmintic marine natural products from sponges.

Compound	Type	Source	Nematode	Toxicity	Country	Reference
Amphilactans A–D	macrolide	*Amphimedon* sp.	*H. contortus*	LD_99_ 0.30–7.5 µg/mL	AUS	[83]
Geodin A	macrolide	*Geodia*	*H. contortus*	LD_99_ 1 µg/mL	AUS	[84]
Onnamide F (2)	polyketide	*Trachicladus laevispirulifer*	*H. contortus*	LD_99_ 2.6 µg/mL	AUS	[85]
Thiocyanatins (1–4)	polyketide	*Oceanapia* sp.	*H. contortus*	LD_99_ 3.1–8.3 µg/mL	AUS	[88]
(−)-echinobetaine A(1)	alkaloid	*Echinodyctium* sp.	*H. contortus*	LD_99_ 83 µg/mL	AUS	[89]
(+)- echinobetaine B(2)	alkaloid	*Echinodyctium* sp.	*H. contortus*	LD_99_ 8.3 µg/mL	AUS	[90]
Phorioadenine A	alkaloid	*Phoriospongia* sp.	*H. contortus*	LD_99_ 31 µg/mL	AUS	[86]
Fromiamycalin	alkaloid	*Monanchora unguiculata*	*H. contortus*	L4 development IC_50_ 26.6 µM; L4 motility IC_50_ 39.4 µM	AUS	[87]
Halaminol A	alkaloid	*Haliclona* sp.	*H. contortus*	L4 development IC_50_ 500 µM; L4 motility IC_50_ 100 µM	AUS	[87]

## Data Availability

Not applicable.
